# Discussion to: Surgically implanted endovascular, microaxial left ventricular assist device: A single-institution study

**DOI:** 10.1016/j.xjtc.2023.10.014

**Published:** 2023-10-16

See Article page 63.

Presenter: Dr Erin M. Schumer

**Dr Frederick Tibayan***(Portland, Ore)*. Congratulations to Dr Schumer and her colleagues on an excellent study. And thank you to the association for the opportunity to discuss it. As the usage of the Impella 5.5 steadily increases, I think these data are going to be very important to help us know what to expect in terms of results for our patients who present with cardiogenic shock or undergo high-risk cardiac surgery. I have two questions. First, outcomes are always heavily influenced by patient selection. Your patients who went under transplant were clearly very carefully selected. Do you think you’ll be able to identify any human dynamic echo or other parameters to help us predict which of our patients with Impella 5.5s are going to well with either transplant-durable left ventricular assist device (LVAD) or a bridge to recovery?

**Figure:**
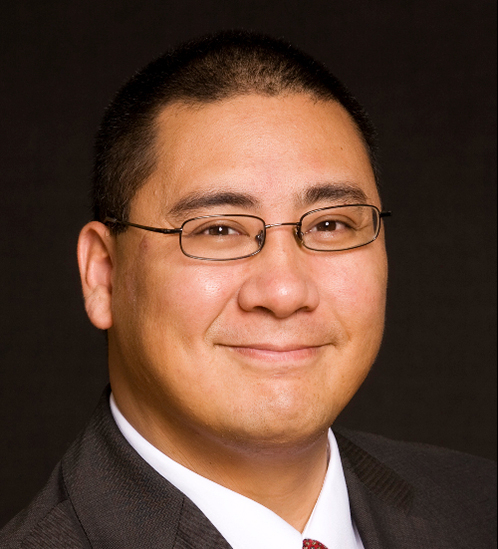

**Dr Erin M. Schumer***(St Louis, Mo)*. Thank you for looking at our paper and giving us these questions. I think there are several things to look at. Our patients for transplant were carefully selected, but 3 of these patients did require extracorporeal membrane oxygenation prior to Impella 5.5, so they were sick patients as well. I think obviously a univentricular failure is going to do better with a durable LVAD than a patient with biventricular failure, who have been our only deaths recently. So, I think looking at bi- versus univentricular dysfunction is going to be the most crucial thing. But again, these patients are all on different modes of support and they’re all really sick, so it just makes it a really difficult decision.

**Figure:**
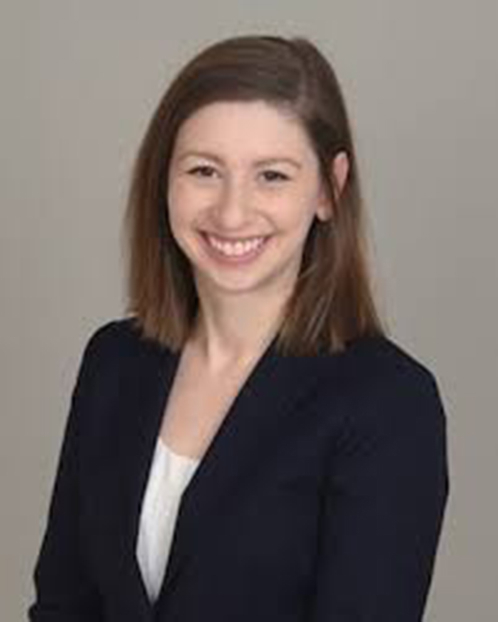

**Dr Tibayan**. Agreed. Second, this is obviously a very challenging patient population. And a lot of patients who present with cardiogenic shock are just not going to do well or be salvageable. Still, the results for 90-day survival are sobering. So, my question is, what can we do or what are you doing at your program to help improve outcomes in these patients?

**Dr Schumer**. I think that we tend to offer these devices pretty liberally even if the situation is pretty grim because we just don’t know the answer to this yet. And so, I think going forward, getting these granular data is going to be really crucial. But, currently, we’re pretty liberal with our use of the Impella.

**Dr Tibayan**. Congratulations on an excellent presentation. And I think it’s to your credit that you recognized your entire team.

**Dr Schumer**. Thank you.

**Dr Craig Selzman***(Salt Lake City, Utah)*. Craig Selzman, Utah. Just to follow up on that a little bit, because you lumped the recovery—in the cardiogenic shock, you lumped recovery and palliation together and that’s a big lump. It’s—

**Dr Schumer**. Agree.

**Dr Selzman**. And so, can you tease that out for us a little bit more? With 50% not around, it just seems like we need to understand that group a little bit more. So, I know you couldn’t probably put it all on a table, but is there a reason why you lumped that, and can you give us any more clarity about that patient group?

**Dr Schumer**. The reason we lumped these categories together is because these are the patients who were not going to be candidates for further therapy. So, I think we felt it was unfair to separate those who died and those who recovered because that—

**Dr Selzman**. The patients mind.

**Dr Schumer**. Yeah.

**Dr Selzman**. Yeah. [laughter] Yeah.

**Dr Schumer**. But they weren’t going to be offered a ventricular assist device (VAD) or a transplant. So, for our study, I think that looking at them as the group that was not offered advanced therapy is a reasonable thing to do, but if you separated them out, our outcomes would look much better. So, I think that’s why we chose to proceed that way.

**Dr Selzman**. Yeah, thanks.

**Dr Slaughter**. Just really quickly, as he’s walking up. So many programs, though, you have to have an exit strategy before you implant it. Because when you say in that group, they weren’t going to be candidates for VAD or transplant. And there’s Dr Selzman and his group at Utah, there’s pretty clear guidelines for who might recover. So, the idea is, they’re not a VAD candidate, they’re not a transplant candidate, and they’re most likely not going to recover, but you put a device in them for wishful thinking?

**Dr Schumer**. Well, I think the option is sometimes—it’s death or give them a shot. And so, it’s hard when somebody has multiorgan failure from an acute myocardial infarction, for instance. It’s difficult to just say, “No. We’re done,” because a lot of times, these patients are salvageable. So, it’s a difficult issue to separate these patients out, but I think it’s a good idea for our paper to look at them separately. But again, we’re very liberal with our use of Impella 5.5 when our patients are sick, and I think it’s better to offer them the chance with support rather than just say no.

**Dr Slaughter**. But I would suggest, though, that this is the group you need to look at, because if they’re still not going to go on to a VAD or a transplant, and quote-unquote, “Then the issue is it’s unlikely they survive the hospitalization,” they're not going to survive long-term, most likely, still.

**Dr Schumer**. Yeah. I think that’s a fair point.

**Diyaar Saeed**. Thank you. Very nice presentation. I am quite surprised by the RVAD implantation rate in your LVAD population. Doing that extracorporeal membrane oxygenation operation when they get durable VAD, that’s in our group, they will get maybe 40% or 50% LVAD. But these are totally different patients than Impella patients. They’re also part of another multicenter study looking at the Impella bridge to durable VADs. And in our multicenter study, which we just submitted, we have like 10% LVAD implantation because the Impella patients are preselected. It doesn’t make a lot of sense to me if you have a patient on Impella, who is living with Impella, having organ function, and goes to operating room, comes out of the operating room with a biventricular assist device. Do you have an explanation for that?

**Dr Schumer**. That’s a great question, and I think a fair criticism of our paper. A lot of these patients aren’t preselected LVADs. Many are coming in shock, and we’re meeting them for the first time. And I don’t remember the number, but a lot of these had a right ventricular assist device (RVAD) prior to the LVAD. So, it would be a biventricular support situation, prior to and not at the time of the durable LVAD. And, additionally, when we first started using the Impella 5.5, I think there’s some learning curve into seeing who the right patient is. And so, our RVAD use has decreased significantly over time. This is a descriptive study, so we didn’t do any comparative statistics, but, for example, over the last year, we’ve only had 3 patients who have required RVAD who were on Impella previously. These RVADs were placed prior to the durable LVAD, so our rate is much lower.

**Dr Slaughter**. Thank you very much.

[applause]

## Conflict of Interest Statement

Dr Itoh is a speaker for Abbott and Abiomed Inc and receives honoraria from both. Dr Kotkar is a speaker for Abiomed Inc. and does not receive honoraria.

The *Journal* policy requires editors and reviewers to disclose conflicts of interest and to decline handling or reviewing manuscripts for which they may have a conflict of interest. The editors and reviewers of this article have no conflicts of interest.

